# Exploring the Impact of a Sleep App on Sleep Quality in a General Population Sample: Pilot Randomized Controlled Trial

**DOI:** 10.2196/39554

**Published:** 2024-08-13

**Authors:** Bianca Tanya Armitage, Henry W W Potts, Michael R Irwin, Abi Fisher

**Affiliations:** 1 Department of Behavioural Science and Health University College London London United Kingdom; 2 Institute of Health Informatics University College London London United Kingdom; 3 Norman Cousins Center for Psychoneuroimmunology, Semel Institute for Neuroscience and Human Behavior, and Department of Psychiatry and Biobehavioral Science, UCLA Geffen School of Medicine University of California Los Angeles, CA United States

**Keywords:** sleep, mobile app, app optimization, intervention, smartphone, general population, mindfulness, cognitive behavioral therapy, CBT, mobile phone

## Abstract

**Background:**

A third of adults in Western countries have impaired sleep quality. A possible solution involves distributing sleep aids through smartphone apps, but most empirical studies are limited to small pilot trials in distinct populations (eg, soldiers) or individuals with clinical sleep disorders; therefore, general population data are required. Furthermore, recent research shows that sleep app users desire a personalized approach, offering an individually tailored choice of techniques. One such aid is Peak Sleep, a smartphone app based on scientifically validated principles for improving sleep quality, such as mindfulness meditation and cognitive behavioral therapy.

**Objective:**

We aimed to test the impact of the smartphone app Peak Sleep on sleep quality and collect user experience data to allow for future app development.

**Methods:**

This was a 2-arm pilot randomized controlled trial. Participants were general population adults in the United Kingdom (aged ≥18 years) who were interested in improving their sleep quality and were not undergoing clinical treatment for sleep disorder or using sleep medication ≥1 per week. Participants were individually randomized to receive the intervention (3 months of app use) versus a no-treatment control. The intervention involved free access to Peak Sleep, an app that offered a choice of behavioral techniques to support better sleep (mindfulness, cognitive behavioral therapy, and acceptance commitment therapy). The primary outcome was sleep quality assessed using the Insomnia Severity Index at baseline and 1-, 2-, and 3-month follow-ups. Assessments were remote using web-based questionnaires. Objective sleep data collection using the Oura Ring (Ōura Health Oy) was planned; however, because the COVID-19 pandemic lockdowns began just after recruitment started, this plan could not be realized. Participant engagement with the app was assessed using the Digital Behavior Change Intervention Engagement Scale and qualitative telephone interviews with a subsample.

**Results:**

A total of 101 participants were enrolled in the trial, and 21 (21%) were qualitatively interviewed. Sleep quality improved in both groups over time, with Insomnia Severity Index scores of the intervention group improving by a mean of 2.5 and the control group by a mean of 1.6 (between-group mean difference 0.9, 95% CI –2.0 to 3.8), with was no significant effect of group (*P=*.91). App users’ engagement was mixed, with qualitative interviews supporting the view of a polarized sample who either strongly liked or disliked the app.

**Conclusions:**

In this trial, self-reported sleep improved over time in both intervention and control arms, with no impact by group, suggesting no effect of the sleep app. Qualitative data suggested polarized views on liking or not liking the app, features that people engaged with, and areas for improvement. Future work could involve developing the app features and then testing the app using objective measures of sleep in a larger sample.

**Trial Registration:**

ClinicalTrials.gov NCT04487483; https://www.clinicaltrials.gov/study/NCT04487483

## Introduction

### Background

Quality sleep is crucial for optimal health [1-3]. However, a third of adults in Western countries report weekly sleep problems, and 30% of adults in the United States report having insomnia [4,5]. Insomnia has been associated with a range of negative health outcomes, including stroke, heart disease, impaired immune function, and poor mental health [1-3]. Therefore, sleep interventions that can be used at a population level are required.

Clinical interventions have demonstrated positive impact on sleep, such as cognitive behavioral therapy (CBT) [6], mindfulness meditation [7,8], and tai chi [9]. However, these can be resource intensive. A possible solution is to use smartphone-based interventions. In the United Kingdom, >79% of adults use smartphones, offering an extremely wide potential population reach [10]. A 2017 systematic review identified 14 telephone-based sleep interventions, 12 of which used smartphone apps [11]. Although the results were promising, the majority were small pilot trials that focused on individuals with diagnosed clinical sleep disorders. The authors provided future research recommendations, including exploring the efficacy of sleep apps in general population samples, analyzing the content of commercially available apps before undertaking interventions, and conducting randomized controlled trials (RCTs) to test efficacy.

Three RCTs of sleep apps explored the impact of CBT for insomnia and reported mixed findings. Horsch et al [12] compared the Sleepcare app (n=74) to wait-list control (n=77) in adults who were experiencing mild insomnia disorders and found a positive impact of the intervention on insomnia severity (Cohen *d*=0.66). In a single-blind RCT of a theory-based sleep app (n=156) versus patient education control (n=156) in participants with insomnia disorder, there was significant improvement in sleep hygiene, sleep quality, and insomnia severity in the sleep app group at 1-, 3-, and 6-month follow-ups [13]. In a wait-list control trial of a sleep app, Refresh, trialed in 371 general population adults who were interested in improving their sleep, insomnia symptoms improved in both intervention and control groups, although more in the intervention group (small effect, Cohen *d*=0.26). However, the group×time interaction was not significant in the overall sample at 3- or 6-month follow-up, and adherence to the intervention and retention at 6 months were relatively low (57%). In addition, only 1 in 3 intervention participants opened the app [14].

### Objectives

The majority of commercially available sleep apps have focused on singular modes of intervention (mainly CBT for insomnia). However, a study, using triangulated results from focus groups (n=9), sleep app reviews (n=434), and a web-based survey (n=167), found that users desire apps to be tailored and adaptable for diverse sleep phenotypes [15]. A newly developed app called Peak Sleep aimed to address this gap by providing a choice of 6 evidence-based techniques: mindfulness meditation, guided imagery, acceptance and commitment therapy, progressive muscle relaxation, relaxation music, and CBT. Although Peak Sleep was based on previous literature on the effectiveness of the aforementioned techniques [16-21], the efficacy of Peak Sleep had not yet been tested.

Therefore, adhering to the Medical Research Council framework for developing and testing complex interventions [22], the aim of this study was to test the app’s efficacy and gather user experience data, allowing for optimization of Peak Sleep.

## Methods

### Design

This was a 2-arm pilot RCT of Peak Sleep developed by Brainbow Ltd compared to a no-intervention control.

### Participants and Recruitment

Participants were recruited via email cascade, social media, and posters on the University College London (UCL) campus. Posters and advertisements indicated that the study was looking for people who wished to improve their sleep and that the study was a collaboration between UCL and Peak Sleep (Brainbow Ltd) testing a sleep app. Participants who registered interest were emailed an information sheet as well as eligibility screening and consent forms on REDCap (Research Electronic Data Capture; Vanderbilt University) software [23,24]. Respondents were eligible if they were residents in the United Kingdom, aged ≥18 years, and owned a smartphone. The exclusion criteria were as follows: respondents diagnosed with a clinical sleep disorder for which they were currently receiving treatment, taking sleep medication ≥1 per week, enrollment on another sleep or lifestyle trial, being unwilling to cease use of any other sleep apps or trackers for the study duration, and being pregnant. The trial was registered on ClinicalTrials.gov (NCT04487483).

### Ethical Considerations

Ethics approval was provided by the UCL ethics committee (16963/001), and all participants provided informed consent. Quantitative data were collected and managed remotely using web-based questionnaires hosted on REDCap [23,24] REDCap directly imports data into the UCL Data Safe Haven portal, which is certified to the ISO 27001 information security standard; conforms to National Health Service Digital’s Information Governance Toolkit; and uses a *walled garden* approach where data are stored, managed, and analyzed within the security of the system. As an incentive, after study completion, all participants received free access to Peak Brain Training (a separate established app developed by the creators of Peak Sleep) for 1 year because Peak Sleep would not be available for users after this study to allow for development based on the findings.

### Randomization and Blinding

After baseline assessments, 1:1 randomization was undertaken by a researcher in the UCL Department of Behavioural Science and Health who was independent of this study using MinimPy (an open-source minimization program for the allocation of participants to groups in randomized trials) [25]. Randomization was not stratified by sleep score (or any other factor) because we did not anticipate baseline differences. After the first participant had been randomly allocated, each subsequent participant was allocated to the trial arm with the lowest imbalance score, with the addition of a 20% random element to reduce the predictability of outcomes. The imbalance score was calculated based on the hypothetical allocation of the next participant to each arm. Limited resource for this pilot study meant that it was not possible to blind the researcher collecting the data (BA) to group allocation.

### Outcome Measures

The time points of study assessments are summarized in Multimedia Appendix 1. Quantitative measurement time points were T0 (baseline, before randomization), T1 (1 month), T2 (2 months), and T3 (3 months). Qualitative interviews were conducted after study completion. Enrollment began on February 13, 2020, and was completed by March 17, 2020; therefore, follow-up data collection periods coincided with the COVID-19 pandemic in the United Kingdom (the country went into full lockdown on March 23, 2020, which was progressively eased from May 11, 2020, onward). Follow-up data collection was completed by June 17, 2020.

### Sleep

The primary sleep outcome was self-reported subjective sleep quality, using the Insomnia Severity Index (ISI) [26] and assessed at T0, T1, T2, and T3. The ISI has been shown to be both reliable and valid when compared with sleep diaries and polysomnography [26]. The ISI has 7 questions, each rated on a 5-point Likert scale (ranging from 0 to 4). The questions cover common sleep issues such as difficulty falling or staying asleep and waking up as well as feelings about current sleep patterns. The sum of the 7 answers yields a total score between 0 and 28, with a higher score indicating worse sleep quality. This measure was selected because the ISI assesses the severity of insomnia symptoms and satisfaction with sleep, as well as interference with daytime functioning, with validated scoring guidelines: no insomnia=0 to 7, subthreshold insomnia=8 to 14, moderate insomnia=15 to 21, and severe insomnia=22 to 28. In addition, it is sensitive to detect changes in insomnia severity in clinical trials that correspond to quantifying minimally important changes in relation to global improvement ratings [27]. As insomnia is the most common sleep complaint in the general population, efforts to adopt a standard research assessment of insomnia have been proposed to allow for comparisons between different studies, including treatment trials, and the ISI is one of the recommended questionnaires for use [28].

A secondary outcome was self-reported subjective sleep, measured using the core Consensus Sleep Diary at T0 and T2. This diary was developed through collaborations with insomnia experts and users [29]. It is a 7-day diary containing 9 questions per day where participants enter the time at which they tried to fall asleep (bedtime); the time taken to fall asleep (sleep latency); the total time awake during the night, referred to as wake after sleep onset (WASO); and the time they woke up for the day and no longer went back to sleep (final awakening).

The original study plan also involved collecting objective sleep data. However, objective assessments had to be canceled due to COVID-19–related lockdown restrictions (we could not access the UCL research center or interact with participants in person, and there was also concern at the time about whether postal data collection could pose an infection risk).

### Intervention

**Figure 1:**
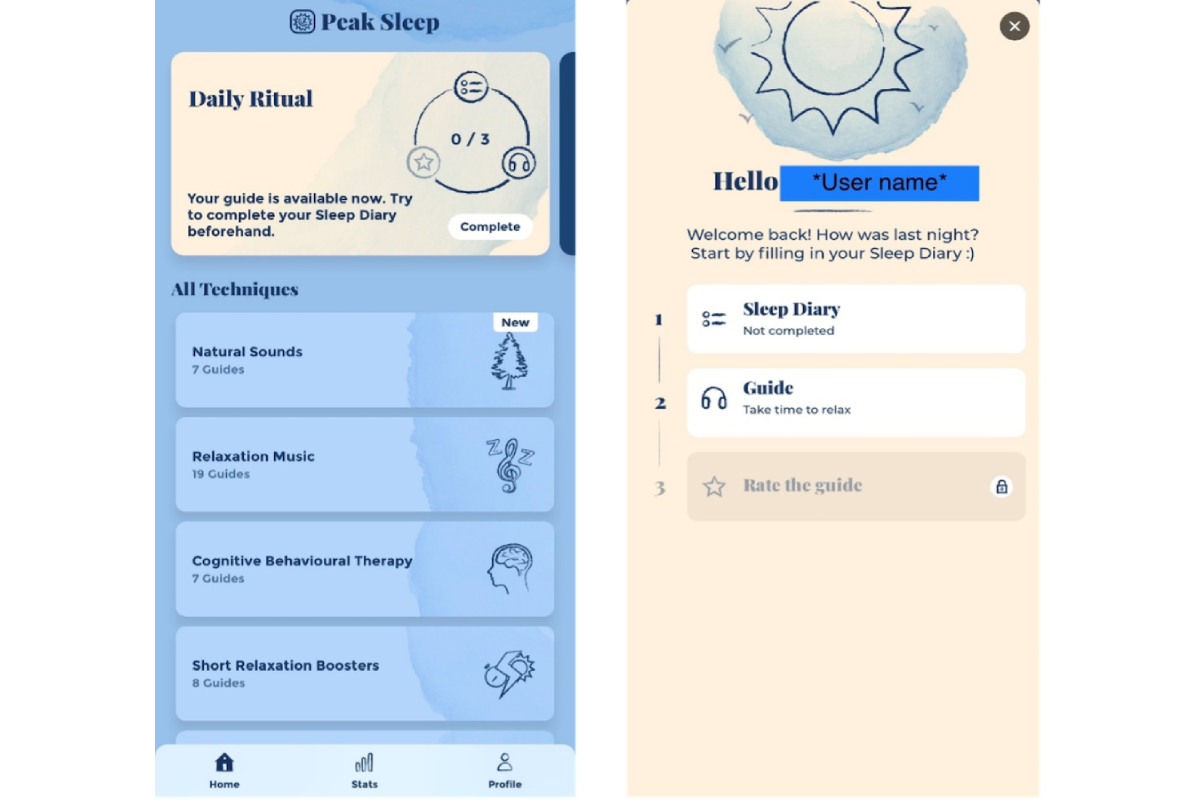
Screenshots of the Peak Sleep app’s “Daily Ritual” feature.

**Figure 2:**
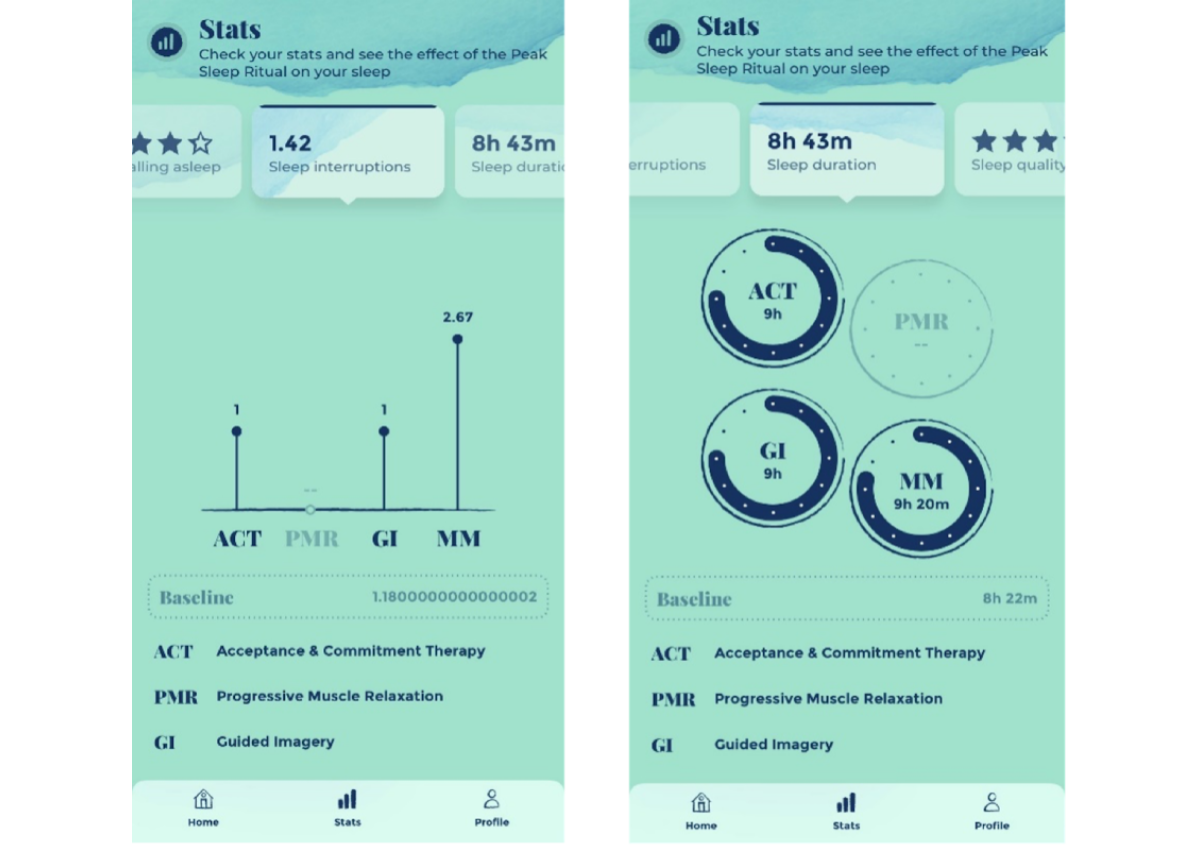
Screenshots of the Peak Sleep app’s “Stats” section.

Intervention participants were given free use of Peak Sleep (Peak Sleep Pro membership) for 3 months. The 3-month duration was selected because the commercial partners felt that this was enough time for users to try all features sufficiently and to indicate some preliminary impact on sleep. In addition, most sleep and behavioral trials incorporated a 3-month follow-up [12-14], allowing a comparison of our findings with those of these trials. Participants were instructed to use the app daily by completing the “ritual” section of the app (Figure 1). This is a daily ritual of (1) a sleep diary rating the previous night’s sleep (to be completed in the morning), (2) listening to a 10-minute audio guide (in the hours winding down to bedtime), and (3) rating the guide. Users were strongly encouraged to complete every step of the ritual, but they could skip step 1 and go straight to step 2 (listening to the guide) if they wanted. However, they could not complete step 3 without completing step 2. The app had an algorithm that took into account the participant’s subjective rating of the guide plus the quality of the subsequent night’s sleep to see what guides worked *best*. Peak Sleep amalgamated this information and used it to recommend future guides. There were multiple guides, each involving a specific technique: relaxation music, CBT, acceptance and commitment therapy, progressive muscle relaxation, guided imagery, and mindfulness meditation. The app also allowed users to override the recommended guide and choose their own. A “stats” section allowed users to see the effect the techniques were having on their subsequent night’s sleep (Figure 2). Other than the aforementioned recommendations for daily use, app-use was self-directed. Participants were asked to inform us if they stopped using Peak Sleep during the trial period and to provide reasons.

### Control

Control participants were aware from the study advertisement and information sheet that the study was testing a sleep app that was a collaboration between UCL and Brainbow Ltd. At the time of testing, the app was not publicly available to download; therefore, participants were not aware of the specific content. Apart from being asked to abstain from using sleep apps or trackers during the trial period and to complete study assessments, controls were assumed to be continuing their usual routines.

### App User Engagement

It was not feasible to access any backend app data in this study due to a change of strategy for the commercial partner after the onset of the COVID-19 pandemic, meaning that limited resource could be directed to this study. Therefore, engagement with the app (intervention group only) was assessed using the Digital Behavior Change Intervention (DBCI) Engagement Scale [30]. Participants completed the DBCI Engagement Scale weekly over the 3 months of the intervention. Participants were asked to rate the extent to which they experienced intrigue, focus, inattention, distraction, enjoyment, annoyance, interest, and pleasure when using the app on a 7-point scale (ranging from *not at all* to *extremely*). The sum of the 8 answers yields a score ranging from 8 to 56, with higher scores indicating higher engagement. For illustrative purposes, engagement scores were categorized as *lower* (scores=8-31), *moderate* (score=32), and *higher* (scores=33-56).

### Qualitative Interviews

Telephone interviews were conducted by BTA with a randomly selected subsample of intervention and control participants who had completed their final study assessments. Interviews were audio recorded and transcribed verbatim. The aim was to recruit approximately 15 to 20 intervention participants and 5 to 10 controls and conduct interviews until it seemed that no new overarching themes were being identified. Interviews were conducted using semistructured topic guides focused on the experience of using Peak Sleep (intervention group) as well as the study procedures and the perceived impact of the COVID-19 pandemic (both groups).

### Analyses

Quantitative analyses were performed using SPSS (version 25.0; IBM Corp). The analysis plan was preregistered on ClinicalTrials.gov (NCT04487483). Sample size was determined partly by what was feasible within the study time frame and resources, but a sample size of 100 participants was considered acceptable based on prior sleep interventions [8,9]. Follow-up rates were reported overall and by randomized group. Engagement with the intervention was summarized to show the patterns of overall engagement and change over time.

To analyze the impact of the intervention versus control on sleep outcomes (ISI and Consensus Sleep Diary outcomes), generalized linear mixed models (GLMMs) were run using the “MIXED” command with full information maximum likelihood estimation (to allow for participants with missing data to be analyzed). This was used to test between-group differences in sleep outcomes throughout the intervention period, adjusting for baseline measures. The model was run with pairwise comparisons. The estimated mean differences are shown, and effect sizes using Cohen *d* with Hedges bias correction were calculated, along with their 95% CIs. Analyses were conducted using intention-to-treat principles. These analyses were exploratory, but effect sizes are reported in line with the CONSORT (Consolidated Standards of Reporting Trials) guidelines for reporting feasibility and pilot studies [31]. As a planned secondary analysis, the level of engagement with the app in the intervention group was included as a covariate to assess whether it had any impact on the findings.

Qualitative data were analyzed using reflexive thematic analysis guided by the 6 phases recommended by Braun et al [32]. AF and BA familiarized themselves with the data by (1) listening to and transcribing the interviews and then reading the transcripts and making notes, (2) coding or recoding the data set and collating codes, (3) generating initial themes from the codes, (4) developing and reviewing themes, (5) refining and naming themes, and (6) writing the results. Due to the word length of the manuscript, overarching themes and subthemes are summarized with illustrative quotes in a table.

## Results

### Participant Characteristics

**Figure 3:**
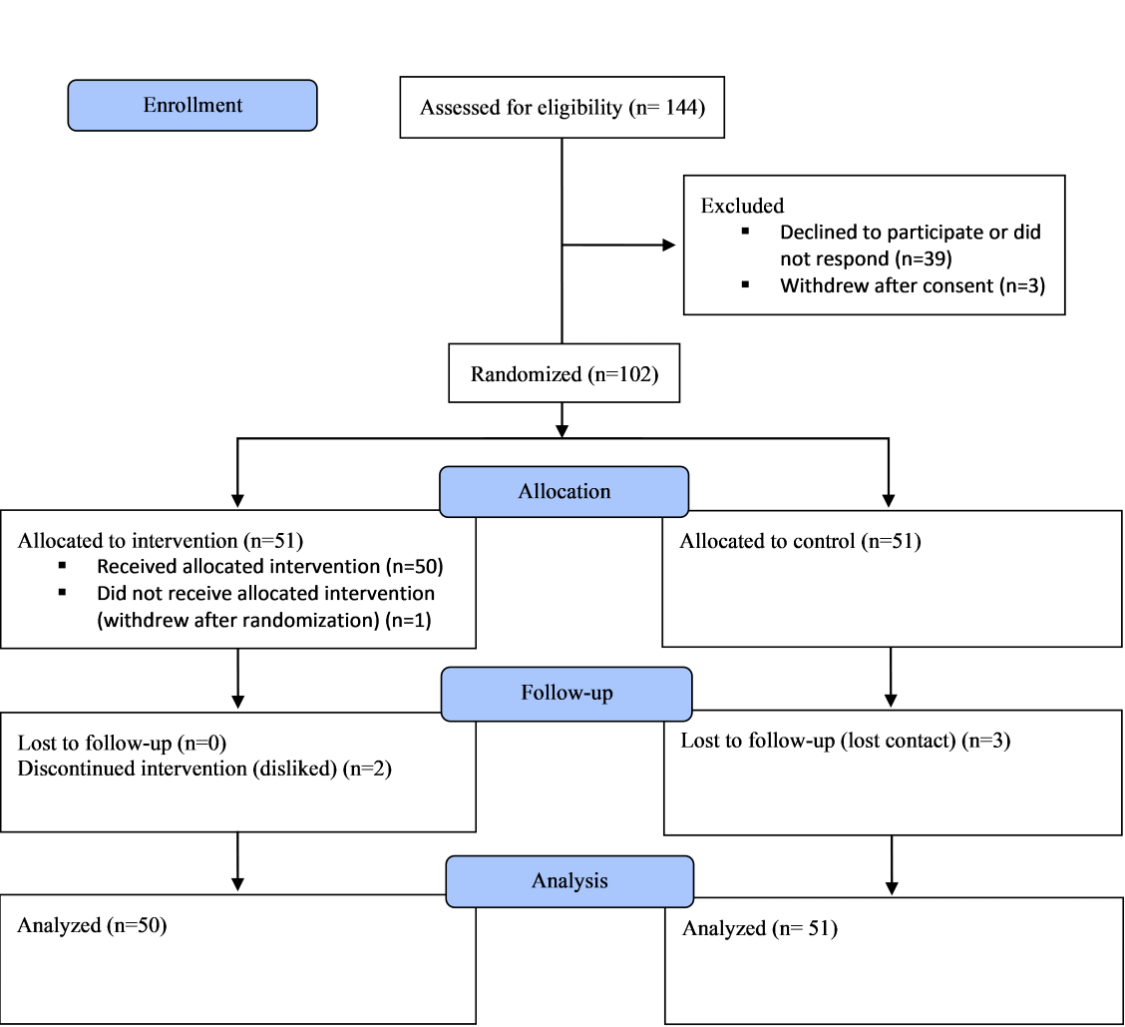
CONSORT (Consolidated Standards of Reporting Trials) flow diagram of participants through the pilot randomized controlled trial of the Peak Sleep app.

Participant flow through the study is summarized in Figure 3. Owing to the methods of recruitment, it was not possible to determine how many people saw the study advertisement. However, 144 individuals expressed interest, and 105 (72.9%) consented. Of these 105 respondents, 3 (2.9%) withdrew after consent; thus, 102 (97.1%) were enrolled and randomized. However, of these 102 participants, 1 (1%) withdrew after randomization, resulting in the inclusion of 101 (99%) participants. All follow-up assessments were completed by 96 (95%) of the 101 included participants. Of the 50 participants who received the intervention, 2 (4%) reported that they stopped using the app before the end of the trial period.

Participant baseline characteristics are summarized in Table 1. Their mean age was 31.0 (SD 11.2) years, and 73% (74/101) identified as female. The mean ISI score at baseline was 10.1 (SD 4.9), indicating subthreshold insomnia. Per the Consensus Sleep Diary, mean WASO was 22.5 (SD 27.0) minutes, mean sleep latency was 22.7 (SD 19.6) minutes, and mean sleep duration was 7.05 (SD 1.01) hours.

**Figure 4:**
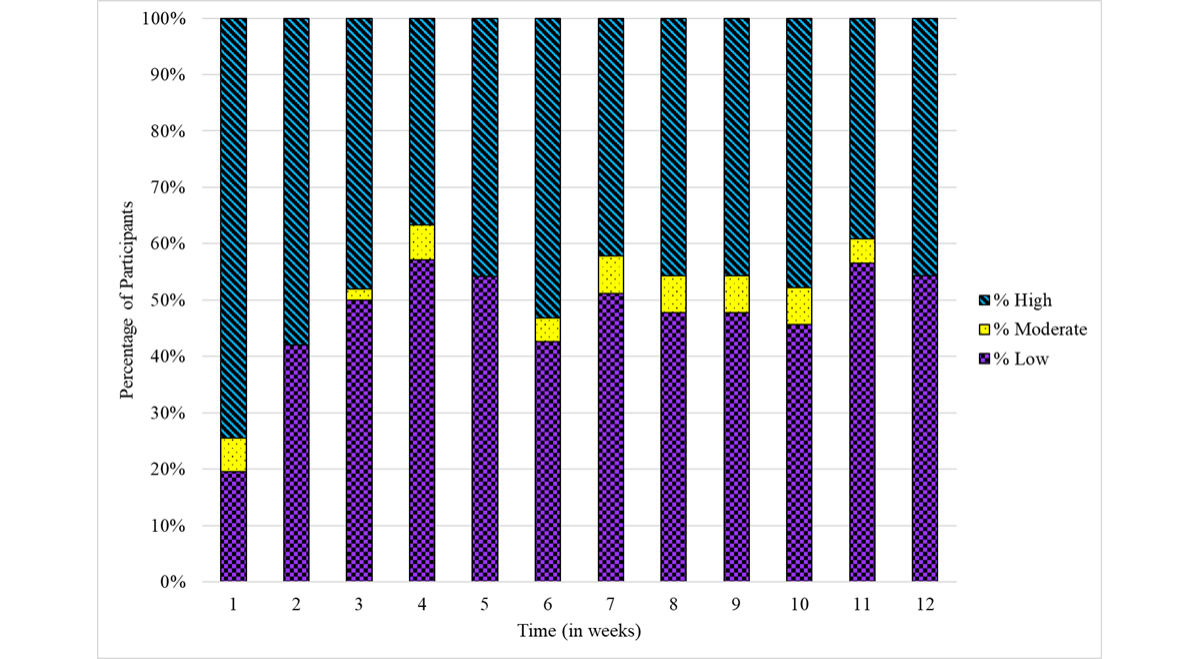
Engagement with the app among intervention participants in the pilot randomized controlled trial of the Peak Sleep app (n=50 for weeks 1-2 and n=48 from week 3 onward). The stacked column chart highlights the percentage level of engagement, measured using the Digital Behavior Change Intervention Engagement Scale [30], after each week of the intervention. Low engagement: score=8 to 31, moderate engagement: score=32, and high engagement: score=33 to 56.

Engagement with the app is presented in Figure 4. Engagement was highest in week 1, and it was relatively stable throughout the rest of the study. From week 2 onward, around half of the sample reported high engagement and half reported low engagement. A very small proportion reported moderate engagement. No adverse effects were reported.

**Table 1:** Participant characteristics at baseline and self-reported sleep variables in the pilot randomized controlled trial of the Peak Sleep app.

		Total (n=101)	Intervention (n=50)	Control (n=51)
**Age (y), mean (SD)**		31.0 (11.2)	33.0 (12.4)	30.0 (9.6)
	Missing, n (%)	1 (1)	0 (0)	1 (2)
**Sex, n (%)**				
	Female	74 (73.3)	36 (72)	38 (74.5)
	Male	27 (26.7)	14 (28)	13 (25.5)
**Ethnicity, n (%)**				
	White	85 (84.2)	41 (82)	44 (86.3)
	Other	16 (15.8)	9 (18)	7 (13.7)
**Education, n (%)**				
	School level	10 (9.9)	4 (8)	6 (11.8)
	Bachelor’s degree	43 (42.6)	23 (46)	20 (39.2)
	Postgraduate degree	1 (1)	0 (0)	1 (2)
	Missing	47 (46.5)	23 (46)	24 (47)
BMI (kg/m^2^), mean (SD)		24.3 (5.2)	23.5 (4.2)	25.1 (6.0)
**Physical activity, n (%)**				
	Sedentary or low active	32 (31.7)	12 (24)	20 (39.2)
	Active or very active	69 (68.3)	38 (76)	31 (60.8)
**Alcohol consumption, n (%)**				
	Once or more than once per week	65 (64.4)	36 (72)	29 (56.9)
	Less than once per week	25 (24.8)	11 (22)	14 (27.5)
	Never	11 (10.9)	3 (6)	8 (15.7)
**Household income, n (%)**				
	Below average	30 (29.7)	15 (30)	15 (29.4)
	Average	20 (19.8)	6 (12)	14 (27.5)
	Above average	32 (31.7)	18 (36)	14 (27.5)
	Missing	19 (18.8)	11 (22)	8 (15.7)
ISI^a^ score, mean (SD)		10.1 (4.9)	11.1 (5.0)	9.1 (4.7)
WASO^b^ (min), mean (SD)		22.5 (27.0)	30.6 (31.7)	14.8 (18.7)
Sleep latency (min), mean (SD)		22.7 (19.6)	23.9 (16.5)	21.5 (22.5)
Sleep duration (h), mean (SD)		7.05 (1.01)	7.05 (1.09)	7.04 (0.94)
Self-awakenings, mean (SD)		3 (2.0)	3 (2.0)	3 (2.0)

^a^ISI: Insomnia Severity Index.

^b^WASO: wake after sleep onset.

### Sleep

**Figure 5:**
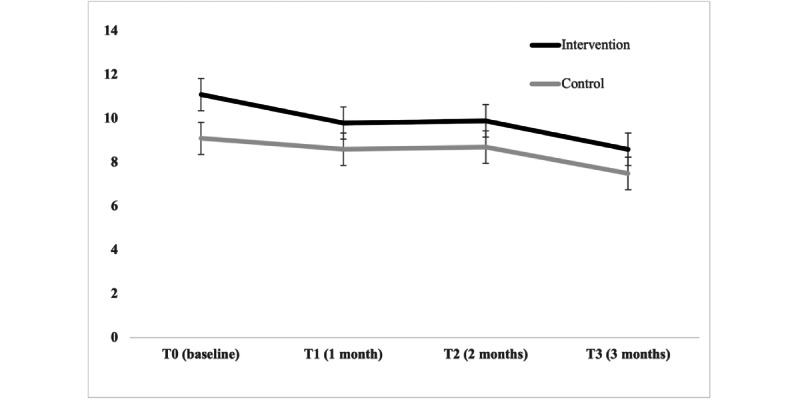
Plot of estimated marginal means of self-reported sleep by group allocation in the pilot randomized controlled trial of the Peak Sleep app. Values are estimated marginal means from a generalized linear mixed model exploring the interaction between group (intervention vs control) and time in 101 participants enrolled in the sleep app study. Group×time interaction was not significant (*P*=.91).

Changes in ISI scores over time by group are presented in Figure 5. The effect of group (*P*=.01) and time (*P*=.04) were significant in the model (the intervention group had slightly higher ISI scores at each time point, and both groups improved over time). However, there was no significant group×time interaction (*P*=.91), suggesting no effect of the intervention. We did not proceed with calculating an effect size because there was no group×time interaction. The intervention group improved over time by a mean of 2.5, while the control group improved by a mean of 1.6 (between-group mean difference 0.9, 95% CI –2.0 to 3.8). Due to the small sample size, the randomization did not achieve balance on the ISI. Therefore, a sensitivity analysis was also performed where the baseline ISI score was included as a covariate; however, there was no difference in the results.

Per the Consensus Sleep Diary, WASO of the intervention group improved by a mean of 9.3 minutes, while that of the control group improved by a mean of 3.0 minutes (Table 2); however, the group×time interaction was not significant (*P*=.65). The sleep latency of the intervention group improved by a mean of 0.46 minutes, while that of the control group improved by a mean of 1.45 minutes (Table 2), but the interaction effect in the GLMM was not significant (*P*=.87). The sleep duration of the intervention group increased by a mean of 0.10 hours, while that of the control group increased by a mean of 0.43 hours (Table 2), but the interaction effect in the GLMM was not significant (*P*=.32).

**Table 2:** Estimated marginal means for primary and secondary self-reported sleep outcomes in the pilot randomized controlled trial of the Peak Sleep app.

Sleep measure			Intervention (n=50)							Control (n=51)							Between-group mean difference
			Baseline, mean (SD)		Follow-up, mean (SD)		Mean change		Baseline, mean (SD)			Follow-up, mean (SD)		Mean change			
**Primary outcome**																	
	ISI^a^ score (range 0-28)	11.1 (5.0)		8.6 (5.5)		2.5		9.1 (4.7)			7.5 (4.7)		1.6		0.9		
**Secondary outcomes**																	
	WASO^b^ (min)	30.6 (31.7)		21.3 (22.8)		9.3		14.8 (18.7)			11.8 (14.3)		3.0		6.3		
	Sleep latency (min)	23.9 (16.5)		23.5 (15.6)		0.46		21.5 (22.5)			20.1 (18.3)		1.45		0.99		
	Sleep duration (h)	7.05 (1.09)		7.15 (1.36)		0.10		7.04 (0.94)			7.47 (1.05)		0.43		0.33		
	Self-awakenings (range 0-7)	3.0 (2.0)		4.0 (3.0)		1.0		3.0 (2.0)			4.0 (2.0)		1.0		0		

^a^ISI: Insomnia Severity Index.

^b^WASO: wake after sleep onset.

### Qualitative Interviews

A total of 21 participants (intervention: n=16, 76%; control: n=5, 24%) were interviewed. The findings are summarized in Textbox 1, with greater detail provided in Multimedia Appendix 2. Views seemed to be polarized: participants either really liked the app or really did not, and it was rare for anyone to offer neutral or ambivalent views. Those who did not like the app cited glitches as off-putting and described the voices used for guides as irritating and enough to make them “switch off.” Key findings from the controls suggested that they felt that completing the diaries regularly made them more mindful and aware of sleep patterns, and they enjoyed the tracking and monitoring. In terms of the impact of the COVID-19 pandemic, although some felt that the pandemic had made their sleep worse (discussing factors such as lower energy expended during the day or higher levels of stress and anxiety), the majority felt that it had not negatively impacted their sleep because they already perceived themselves as bad sleepers before the pandemic. Some reported that the removal of the stress of morning commutes when working from home, as well as socializing later at night, meant that they felt that their sleep actually improved (less requirement for alarms or removal of the pressure of commuting). Only 2 (12%) of the 16 intervention participants reported that the COVID-19 pandemic had impacted their interaction with the app (n=1, 50% negatively; n=1, 50% positively). All participants expressed extreme gratitude for sleep research and talked about how they felt that there was a lack of research in general population poor sleepers.

**Summary of qualitative quotes from the thematic analysis of 21 participants (intervention: n=16, 76%; control: n=5, 24%) in the pilot randomized controlled trial of the Peak Sleep app.**

**Factors negatively influencing enjoyment of the app**

- Technical glitches
  “The glitches really irritated me. There was one glitch where if I tried to switch to a different guide, I would still have to rate the original recommended guide even though I hadn’t listened to it. Some nights when I didn’t do a session it would still tell me I had to fill out the diary the next morning which irritated me because I had to fill out nonsense.”
- Voices of guides
  “Some of the voices weren’t the most relaxing which is distracting when you’re trying to get to sleep.”
  “The voices weren’t very soothing or appropriate for the guides themselves.”

**Preferred app features**

- Muscle relaxation and mindfulness meditation
  “Muscle relaxation. I suffer from headaches and focusing on relaxing the body really helped.”
  “Mindfulness Meditation one would cause me to fall asleep before the guide finished so that worked too.”
- Concise guides
  “It didn’t draw on too long, liked that it was concise and well put together.”

**Perceived benefits**

- Establishing a routine or habit
  “It allowed me to set up a new routine for myself and helped me relax. It made it very easy to create a habit. Liked that it wasn’t a chore to fill out the morning diary, was quick and easy to do.”
- Tracking or self-monitoring of sleep
  “I quite enjoyed having to think about my sleep and be super aware of it.”

**Missing features**

- Linking with objective measure of sleep
  “Introduce some objective markers of sleep quality because the self-report ones are biased to how I was feeling at the time, especially during a pandemic.”
  “[A] sleep tracking feature using accelerometery to see which sessions had the best objective impact on sleep quality.”
- Increased diversity or choice
  “Guides to be a bit more diverse as they tend to be a bit repetitive within the categories.”

**Perceived improvements in sleep due to app**

- Improved sleep
  “Yes, I felt like I was getting better sleep when using it. More relaxed.”
- Had not improved sleep
  “Personally, for me, I don’t think so. I think that it may help some people, but I would say that it didn’t necessarily improve my sleep at all.”
- Unsure
  “I don’t know because I find it hard to tell. It maybe improved the amount of times I wake up during the night but not how long it took me to fall asleep.”

**Impact of the COVID-19 pandemic**

- Impact on sleep
  “Terribly. I work from home anyway, but it feels like it’s disrupted any semblance of routine that I had. It’s been really difficult to get that back, so I find myself not being as in control of my sleep schedule. It’s obviously very stressful as well—with my ability to fall asleep and stay asleep if I wake up in the night, it can be quite disruptive.”
  “Initially sleep got worse. But long term my sleep was possibly a bit better because I had more time to sleep more.”
  “I would hazard to say not at all. Had a few worried nights but I don’t think it’s made my bad nights any worse than they already were.”
- Impact on perception of the app
  “Probably negatively affected it as I was quite irritable when using Peak Sleep. Harder to integrate using the app into daily life due to lack of routine. It was the only thing I had to do each day so if I would forget I’d get pissed off that I still had something I had to do before I could go to bed.”
  “Don’t think it had a negative effect. Was nice to have something to look forward to in my routine.”
  “Not very much. Found it easier to stick with using the app because work didn’t get in the way and could focus more on the app as something fixed to do that day.”

## Discussion

### Principal Findings

This pilot RCT explored the acceptability and early efficacy of the Peak Sleep app. Adherence to study procedures was high, whereas experience of the intervention was mixed. Our primary outcome (change in sleep quality) suggested no significant effect of the intervention, and none of the secondary sleep measures demonstrated any impact of the intervention. After the first month follow-up, both groups showed very similar improvements in sleep scores.

The most comparable study was an RCT of a CBT-based sleep app [14]. Similar to our study, the authors included general population adults who wanted to improve their sleep. In line with our study, there were improvements in sleep in both intervention and control groups and no group×time effect in the overall sample. However, in a subanalysis with individuals who had poor sleep at baseline, there was a positive impact of the intervention. These results were not available when we were designing our study; therefore, we did not plan any subanalyses by sleep scores, and our sample was smaller, meaning that this was not feasible. Indeed, in 2 other RCTs that only included participants with insomnia disorders, there were significant positive effects of sleep apps [12,13]. Taken together with the findings of our study, this does bring into question whether trials of sleep apps should be restricted to those who experience threshold sleep problems. However, in these trials, one wants to test the app in the target users as far as possible; therefore, unless apps are marketed as being effective only for those with sleep problems, this is problematic. In future, researchers should at least plan to build in a subanalysis of those experiencing sleep problems.

Aside from the fact that we did not explore findings in a subgroup of poor sleepers only, there are a number of other reasons the app may have demonstrated no impact on sleep in our study. The app simply may not have been effective. However, we should be cautious about interpreting a negative result in a pilot study. The sample size was relatively small (n=50 in the intervention group and n=51 in the control group). Our target sample size was based on previous sleep studies that had been powered to detect effects [8,9]. It should be noted that these studies tested more intensive face-to-face interventions and yielded larger effect sizes. However, our study was sensitive enough to detect significant improvements in sleep over time in both groups, and there was no indication of positive trends in favor of the intervention. Reported engagement with the intervention was mixed, and qualitative interviews suggested that the app was polarizing in terms of content. It is somewhat unsurprising that people who did not like the app content might not benefit. In the aforementioned RCT [14], 1 in 3 participants did not even open the app.

In addition, in line with this RCT, in our study, improvements in self-reported sleep over time were seen in both intervention and control participants. The simple act of partaking in a sleep trial could make participants more aware of their sleep habits. Minor improvements to sleep hygiene due to heightened mindfulness of their sleep pattern could have been driving the improvement seen. Indeed, a key theme from the qualitative interviews of control participants suggested that sleep diaries made them more aware of their sleep. In fact, tracking and self-monitoring are important behavior change techniques in other contexts and perhaps for sleep too. However, this does highlight the need for objective device-based assessments. The original study plan had been to incorporate these, but the onset of the COVID-19 pandemic meant that this was not feasible.

The flyers for the study specified that it was a sleep study; therefore, individuals possibly volunteered for the study when their sleep was worse, but then it might have improved over time because of regression to the mean. In addition, the impact of the COVID-19 pandemic may have had an influence. Within a month of participant enrollment, the United Kingdom went into lockdown, and many people were furloughed, lost their jobs, or had to adapt to working from home. Such an abrupt change of routine, in addition to anxiety over their own health and that of others, may have caused disruption. However, while qualitative interviews supported the notion that many participants did experience higher levels of anxiety, participants reflected that they were already poor sleepers at baseline and did not perceive their sleep as getting worse as a result.

The findings from the secondary outcomes, with respect to change in sleep quality, supported those from the primary outcome. Change in WASO, sleep latency, or sleep duration did not differ by group, reinforcing the finding that Peak Sleep had no impact on sleep quality. However, interestingly, when overall engagement score in the intervention group was run as a covariate on the aforementioned analyses, the results were also not significant. A common cause for intervention ineffectiveness in digital health is a lack of engagement with the intervention [33]. We saw no interaction by engagement scores and sleep, suggesting that those who engaged with the app did not show more of an improvement in any of the sleep measures than those who did not engage. These results should be viewed with caution because our sample was relatively small, particularly for the subanalyses, and we did not have backend user data; instead, we relied on self-reported engagement.

### Acceptability and Optimization of Peak Sleep

It is important to consider constructive suggestions to improve the app for those whose tastes it does align with. Other than fixing the technical glitches mentioned, the common theme of interviewees suggesting that those voicing the guides should undertake an element of voice training could be an explanation for the lack of efficacy of the app on sleep quality. Although those voicing the guides in Peak Sleep were all trained professionals in their field, they are undoubtably used to the more conventional, face-to-face method of delivering their intervention. There is, expectedly, an element of the experience lost when adapting to solely audio output, and perhaps an exploration of voice training from a professional voice actor might provide the skills to compensate for this. Further suggestions regarding enhancing the tailored nature of Peak Sleep, such as adding a notes section or accelerometery technology, indicated that the tailored aspect of the app was encouraged and well received. This ties into previous literature communicating the demand for more personalized approaches [15]. Therefore, Peak Sleep seems to meet demand in this regard but requires further work to perfect this feature.

There is recognition in the literature that there should be more RCTs of app effectiveness, but there are a variety of challenges to achieving this [34,35]. Health technology companies do not have the same cultural familiarity with RCTs as the pharmaceutical sector and should be applauded for engaging in such research. The RCT, like all technology evaluations, can be part of an iterative cycle of improvement [36]. Regarding our RCT, the company involved had already planned to withdraw the availability of the app to allow for improvements to be made in response to the trial results.

### Strengths and Limitations

In this study, we successfully recruited our target sample and had very low dropout rates. We believe that our low dropout rates may have been due not only to emphasis at enrollment on the importance of both intervention and control arms and of retaining participants whether they liked the app or not but also to the onset of the COVID-19 pandemic, meaning that because most of our participants were in lockdown at home, some of the usual barriers to participation were removed. Participants were also incentivized with access to the well-established Peak Brain Training app on completion of the study. This was a moderately sized trial and was not powered to detect small effects. We were powered to detect a medium effect size, which is the usual relevant effect size for clinical sleep intervention research [9]. In addition, although our sample was predominantly female, sleep problems are most prevalent in women [37]; therefore, we believe that our sample was reflective of the general population’s need for sleep aids.

However, there were limitations to our study. First, the COVID-19 pandemic unexpectedly spanned the entire duration of the RCT and, as discussed, seems to have had an effect on some participants’ sleep. In addition, as revealed by the intervention participants in the qualitative interviews, the pandemic had a mixed effect on their feelings toward Peak Sleep. The increase in anxiety brought on by the pandemic seemed to manifest itself as either increased irritation toward the app or increased demand for it for some. This could seem to cancel out but, alternatively, could be responsible for the equal split of engagement, depending on the participant’s personal reaction to the global stressor of the pandemic. Although suggestions for app optimization are no doubt still valid, once these optimizations have been made, further research should ascertain engagement with the app free from the influence of a global pandemic. We did not control for the use of previous sleep apps. However, we wanted to capture the experience of potential real-world general population users, who may have used other sleep apps in the past. We also expected that the randomization would balance potential confounders across groups.

The lack of an objective measure of sleep (access to the research center and mail-outs were not permitted because of COVID-19–related restrictions) was also an unforeseen limitation. If objective data had indicated an improvement in sleep quality, then the notion of a lack of group effect may have been challenged because there are often discrepancies between objective and subjective measures. Overall, although objective data would have undoubtedly enhanced the data, the primary outcome of the ISI is a reliable measure, and it is unlikely that objective data would have challenged this dramatically.

Despite requesting app use data from the commercial partner, access was ultimately not possible. While these data would have been very useful, in our intention-to-treat approach, the volume of use would not have impacted our main findings. However, it would certainly have enhanced our understanding of user engagement with specific features. We attempted to address this by collecting DBCI data and qualitatively interviewing participants, and both these assessments were reflective of issues around engagement as well as app quality and use, which could explain the limited impact on sleep. However, future studies should endeavor to secure backend data where possible.

In our study, we used a control group who were not offered an *attention control*. Therefore, we were not controlling for attention and exposure to an app. In our study, we did not observe an effect of the intervention, suggesting that exposure to the app was not adding anything. However, if we had, it could have undermined the findings to not have attempted to control for app exposure. Therefore, future studies should carefully consider what to offer the control participants to account for exposure to an app. We had a relatively short follow-up. This was partly due to time and funding constraints. However, we viewed this trial as a pilot in the hope of using the data to improve the app and then exploring the possibility of applying for funding for a larger trial with longer follow-up and objective measures.

One final limitation was the wording of the Consensus Sleep Diary questions. Of the 101 participants, 5 (5%) misunderstood the question “What time was your final awakening?”; they believed that it referred to their last nighttime awakening and not the final time they woke up in the morning and no longer returned to sleep. The misinterpretation of “final awakening” had a knock-on effect on the questions “How many times did you wake up, not counting your final awakening?” “In total, how long did these awakenings last?” This meant that both sleep duration and WASO could not be interpreted from these participants. In addition, 3 (3%) of the 101 participants seemed to have misinterpreted the question “What time did you try to go to sleep?” because they provided an earlier time than their answer to the question “What time did you get into bed?” This meant that sleep duration could not be calculated for these participants as well. Generally, however, most participants interpreted the questions correctly, and only a small amount of sleep duration and WASO data were missing for this reason. Future research could accompany the sleep diary with further clarification of these terms to avoid misinterpretation.

### Conclusions

In this trial, self-reported sleep improved over time in both intervention and control arms, with no impact by group, suggesting no effect of the sleep app. Qualitative data suggested polarized views on liking or not liking the app, features that people engaged with, and areas for improvement. Future work could involve integrating popular features into the app and then testing the app using objective measures of sleep in a larger sample.

## Appendix

Multimedia Appendix 1 Time points of study assessments. A flow diagram representing participants’ chronological course through the study and the time points of assessments. Oura Ring (Ōura Health Oy) follow-up (dark green box) was canceled due to COVID-19-related lockdown restrictions.

Multimedia Appendix 2 Qualitative report.

Multimedia Appendix 3 CONSORT-eHEALTH checklist (V 1.6.1).

## Abbreviations

CBT: cognitive behavioral therapy
CONSORT: Consolidated Standards of Reporting Trials
DBCI: Digital Behavior Change Intervention
GLMM: generalized linear mixed model
ISI: Insomnia Severity Index
RCT: randomized controlled trial
REDCap: Research Electronic Data Capture
UCL: University College London
WASO: wake after sleep onset

## Data Availability

The data sets generated and analyzed during this study are stored in an encrypted database in the UCL Data Safe Haven in line with our data protection and ethics approval requirements. Data may be made available by the corresponding author on reasonable request.
