# Supplementary figures and images for: Exploring the Impact of a Sleep App on Sleep Quality in a General Population Sample: Pilot Randomized Controlled Trial
Source: JMIR Form Res. 2024 Aug 13;8:e39554. doi: 10.2196/39554 (PMC11350301; doi:10.2196/39554)

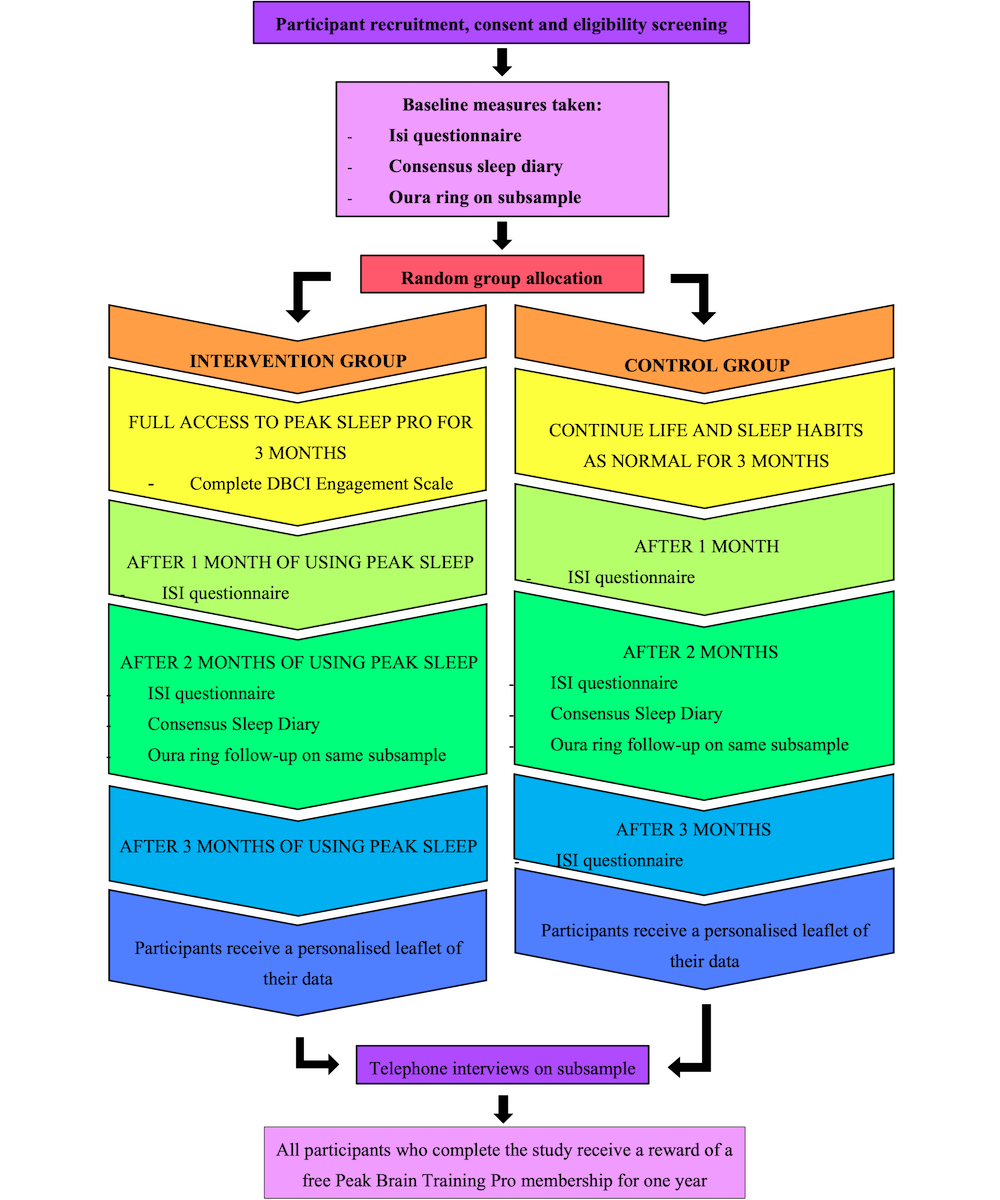

Supplement: Multimedia Appendix 1 [file formative_v8i1e39554_app1.png]
