# Supplementary material for: Exploring the Impact of a Sleep App on Sleep Quality in a General Population Sample: Pilot Randomized Controlled Trial
Source: JMIR Form Res. 2024 Aug 13;8:e39554. doi: 10.2196/39554 (PMC11350301; doi:10.2196/39554)
Supplement: Multimedia Appendix 2 [file formative_v8i1e39554_app2.docx]

**Supplementary material – Qualitative Report**

**Factors negatively influencing enjoyment of the app**

Some participants were very positive about the app and most reported enjoying use the app to some extent. However, the majority reported mediocre responses like “so, so” or “it was OK”. The factors that most strongly related to how positively the app was viewed were glitches and voices of guides.

***Technical glitches***

Those who had mediocre, or negative responses reported consistent glitches that frustrated them. A particular glitch that was raised frequently related to have to predefine a set bedtime (when in reality they can be variable), and not being able to accurately input sleep times, or having to rate guides that they hadn’t actually completed:

*“The glitches really irritated me. There was one glitch where if I tried to switch to a different guide I would still have to rate the original recommended guide even though I hadn’t listened to it. Some nights when I didn’t do a session it would still tell me I had to fill out the diary the next morning which irritated me because I had to fill out nonsense.”*

***Voices of guides***

In addition, a number participants reported that they found the voices leading the guides weren’t very soothing, which they viewed as being at odds with the content and nature of the intervention. This strongly influenced their overall enjoyment of the app.

*“Some of the voices weren’t the most relaxing which is distracting when you’re trying to get to sleep”*

*“The voices weren’t very soothing or appropriate for the guides themselves.”*

**Preferred app features**

***Muscle relaxation and mindfulness meditation***

A number of the guides were commonly mentioned by name as being preferred. These were Progressive Muscle Relaxation and Mindfulness Meditation.

***Concise guides***

The majority of participants reported that they liked the length of the guides, perceiving them as short and feasible to build into daily routine. Although two participants would have preferred them to be longer.

**Perceived benefits**

***Establishing a routine/habit***

A number of participants discussed throughout the interviews that being encouraged to use the app helped them establish a bedtime routine, and whether they particularly engaged with the specific app features or not they felt this was a beneficial outcome, and something they would continue even if they didn’t continue to use the Peak Sleep app.

***Tracking/self-monitoring of sleep***

Encouragement to track or monitor sleep was also a perceived benefit of being in the intervention. If anything, participants wished that the app would encourage more of this. Controls also commonly mentioned that they had really enjoyed completing the diaries and monitoring of sleep.

*“I quite enjoyed having to think about my sleep and be super aware of it.”*

**Missing features**

Throughout interviews participants discussed that there were features they would have liked to have seen, or seen amended.

***Linking with objective measure of sleep***

A number of participants suggested that including links with a direct measure of sleep, rather than reliance on inputting sleep times would strengthen the app and enhance self-monitoring/help them see what was working best for them.

*“Introduce some objective markers of sleep quality because the self-report ones are biased to how I was feeling at the time, especially during a pandemic.”*

*“..a sleep tracking feature using accelerometery to see which sessions had the best objective impact on sleep quality.”*

***Increased diversity / choice***

Participants noted that the app guides became repetitive quickly and increasing the number of options to choose from would strengthen the app.

**Perceived improvements in sleep due to app**

***Split three ways***

Around a third of participants thought it had improved their sleep and a third felt strongly that it hadn’t.

*“Yes I felt like I was getting better sleep when using it. More relaxed.”*

*“Personally, for me, I don’t think so. I think that it may help some people but I would say that it didn’t necessarily improve my sleep at all.”*

It was also very common for people to be unsure, saying things like ‘I think so’ or ‘I don’t know’ or to acknowledge that they hadn’t used it as regularly as was recommended or to say it helped on some nights but not others, or didn’t help at the start, but felt it did towards the end of the study once they became more familiar with the features and stats. Some participants felt that it improved certain aspects of sleep, but not all.

*“I don’t know because I find it hard to tell. It maybe improved the amount of times I wake up during the night but not how long it took me to fall asleep.”*

Although it was often those who didn’t enjoy the app that perceived no impact on sleep this was not always the case (sometime people enjoyed aspects of the app despite not perceiving improvements in their sleep).

**Impact of COVID-19**

***Impact on sleep***

Around half of the participants felt as though the pandemic negatively affected their sleep, mostly due to increased stress and disruption of routine. A couple of these participants felt as though their sleep later improved despite initially getting worse, due to adjusting to having more time and less responsibilities.

*“Terribly. I work from home anyway, but it feels like it’s disrupted any semblance of routine that I had. It’s been really difficult to get that back, so I find myself not being as in control of my sleep schedule. It’s obviously very stressful as well – with my ability to fall asleep and stay asleep if I wake up in the night, it can be quite disruptive.”*

*“Initially sleep got worse. But long term my sleep was possibly a bit better because I had more time to sleep more.”*

Many participants felt as though it had no impact because they felt their sleep was bad anyway.

*“I would hazard to say not at all. Had a few worried nights but I don’t think it’s made my bad nights any worse than they already were.”*

***Impact on perception of the app***

Around half of the participants felt that the pandemic did not influence their feelings about Peak Sleep at all. The other half had mixed views, with some leaning towards disliking having to use it due to feeling generally worse and finding it difficult to integrate into their lives due to a lack of other routine, and some appreciated having something to look forward to that allowed them to create a routine, but mostly participants didn’t feel as though the pandemic strongly affected their feeling towards the app.

*“Probably negatively affected it as I was quite irritable when using Peak Sleep. Harder to integrate using the app into daily life due to lack of routine. It was the only thing I had to do each day so if I would forget I’d get pissed off that I still had something I had to do before I could go to bed.”*

*“Don’t think it had a negative effect. Was nice to have something to look forward to in my routine.”*

*“Not very much. Found it easier to stick with using the app because work didn’t get in the way and could focus more on the app as something fixed to do that day.”*
